# 청소년의 디지털 성 미디어 리터러시 교육 중재 연구 동향: 2015–2025 스코핑 리뷰

**DOI:** 10.4069/whn.2026.05.26

**Published:** 2026-06-30

**Authors:** Gayoung Kim, Hae Won Kim

**Affiliations:** 1 College of Nursing, Seoul National University, Seoul, Korea; 1 서울대학교 간호대학; 2 Center for Human-Caring Nurse Leaders for the Future by Brain Korea 21 (BK 21) Four Project, Seoul National University, Seoul, Korea; 2 서울대학교 BK21 사업; 3 The Research Institute of Nursing Science, Seoul National University, Seoul, Korea; 3 서울대학교 간호과학연구소

**Keywords:** Adolescent, Information literacy, Mass media, Scoping review, Sex education

## Abstract

**Purpose:**

This scoping review analyzed research trends and the effectiveness of sexual media literacy educational interventions for adolescents and provided foundational evidence for developing effective programs.

**Methods:**

Guided by Arksey and O’Malley’s scoping review framework, ten databases were systematically searched for studies published between 2015 and 2025. Studies involving adolescents and individuals who educate or mediate adolescents’ media use were included. Two independent reviewers screened titles, abstracts, and full texts, yielding a final sample of 11 studies.

**Results:**

The included studies predominantly targeted middle school students, and the theory of planned behavior was the most commonly applied theoretical framework. Most programs (81.8%) emphasized strengthening adolescents’ ability to interpret sexual content in media. Program duration ranged from 1 to 10 sessions, with total instructional time ranging from 90 minutes to 18 hours. Reported outcomes included improvements in sexual media literacy (72.7%), knowledge (36.4%), attitude (54.5%), behavior (45.5%), belief (18.2%), and interpersonal process (36.4%). The main teaching methods were lecture-based delivery (63.6%), offline interactive activities (63.6%), and web-based programs (36.4%). Overall, the programs improved adolescents’ sexual media literacy, sexual health outcomes, and sexual health-related communication skills.

**Conclusion:**

This review provides evidence that sexual media literacy programs can improve adolescents’ media interpretation skills and sexual health-related outcomes. Integrating learner-centered approaches with media-based instruction may be particularly useful and has meaningful implications for developing tailored sexual media literacy programs for adolescents.

## Introduction

2000년대 이후에 출생한 청소년들은 어린 시절부터 인터넷과 모바일 환경에 노출되며 성장한 세대로, 다양한 디지털 플랫폼에 능숙한 ‘디지털 원주민(digital native)’으로 불린다[1-3]. 실제로 한국의 10대 청소년의 주요 정보 획득 수단은 스마트폰, 태블릿 PC 등을 통한 인터넷이며, 성 관련 정보 역시 전통적인 면대면 교육보다 유튜브, SNS (Social Network Service), 포털사이트 등 디지털 기반의 온라인 매체를 통해 주로 획득하고 있다[4,5].

최근 디지털 기술과 인공지능 기반 플랫폼의 발전은 청소년의 미디어 이용 환경과 정보 탐색 방식을 확장했으며, 알고리즘 기반 개인화 추천 시스템은 성 관련 콘텐츠 노출의 범위와 양상을 변화시키고 있다[6,7]. 이는 청소년들에게 정보 접근성을 높이는 긍정적인 측면도 있지만, 동시에 선정적이고 자극적인 성 콘텐츠에 무분별하게 노출될 위험성을 동반한다. 특히 콘텐츠 제작과 유통이 조회 수 기반의 수익 구조에 의존하는 현 온라인 미디어 환경에서 청소년들은 무분별하게 유통되는 음란물, 왜곡된 성 인식, 성차별적 표현에 더욱 쉽게 접근할 수 있다[8]. 청소년기는 인지 및 도덕 판단능력이 아직 충분히 발달하지 않은 시기로, 이러한 유해 콘텐츠는 왜곡된 성 인식 및 성 행동으로 이어질 위험이 있으며, 실제로 최근 딥페이크, 불법 촬영물 유포 등 디지털 성범죄에 10대 청소년이 가해자로 연루되는 사례가 증가하고 있다[9,10].

디지털 환경에서 유해한 성 콘텐츠로부터 청소년들을 보호하기 위해서는 단순한 정보의 차단을 넘어 윤리적 판단력을 갖추고 스스로를 통제할 수 있는 역량을 키워줄 수 있어야 한다[11]. 따라서 디지털 전환의 가속화를 경험하며 살아가는 현대 청소년 성교육에서는 미디어의 내용들을 수동적으로 받아들이기보다 능동적으로 해석하고 비판할 수 있도록 하는 디지털 성 미디어 리터러시(digital sexual media literacy)가 필수적이다[10,12]. 특히 코로나 팬데믹을 계기로 디지털 기술 활용이 더욱 확산되는 가운데, 우리 사회에서는 청소년 학생들의 디지털 기기 사용 증가와 리터러시 저하를 우려하는 목소리가 높아져 왔다[13]. 리터러시는 문자를 읽고 쓰는 능력에서 특정 분야에서 활용되는 역량과 지식이라는 의미로 확장되면서, 이에 대한 연구도 증가하고 있다[14]. 경제협력개발기구(Organization for Economic Cooperation and Development, OECD)에서 발간한 ‘21세기 독자’ 보고서에서 한국 학생들은 디지털 환경에서 사실과 의견을 구분하는 능력인 디지털 리터러시가 OECD 국가 중 꼴찌에 가까운 수준으로 보고되었다[15]. 이에 따라 2022년 3월 25일 시행된 ‘디지털 기반의 원격교육 활성화 기본법’에서는 ‘디지털 미디어 문해(리터러시) 교육’을 제도화하기에 이르렀다[16].

디지털 미디어 리터러시란 미디어, 디지털, 정보를 포괄하는 개념으로[17], 미디어에 접속하는 능력뿐 아니라 정보를 해석하고 이해하며, 분석 및 평가를 통하여 자신의 생각과 견해를 표현하는 능력을 말한다[18,19]. 디지털 미디어 리터러시에 성이라는 교육 내용을 더한 개념인 디지털 성 미디어 리터러시는 청소년이 디지털 환경에서 접하는 성 관련 정보를 비판적으로 해석하고 유해한 콘텐츠를 분별하여 올바르게 수용할 수 있는 역량으로, 청소년의 건강한 성 인식과 성적 자율성을 형성하고 위험 행동을 예방하기 위한 핵심적인 요소로 간주된다[20,21].

선행 연구들은 우리나라 성교육이 주로 생물학적 지식 전달에 초점을 맞추고 있으며, 디지털 미디어 환경에서 필요한 비판적 해석 능력과 정보 선별 능력을 체계적으로 교육하지 못하는 한계를 지적하고 있다[11,21]. 반면, 미국이나 유럽 등의 국가에서는 온라인 개인정보 보호, 성차별적 콘텐츠 비판, 사이버 폭력 예방과 같은 디지털 성 미디어 리터러시 성교육을 정규 교육과정에 통합하여 체계적으로 교육하고 있다[22]. 이에 따라 최근 우리나라에서도 디지털 성 미디어 리터러시 교육에 대한 논의가 증가하고 있으나 아직 이에 대한 제도적 기반이나 체계화된 교육 프로그램은 미비한 실정이다[23].

청소년들의 눈높이에 맞는 체계적인 디지털 성 미디어 리터러시 교육 프로그램 개발을 위하여 먼저 현황 파악이 필수적이다. 2000년대 들어 국내에서 리터러시 연구에 대한 관심은 급격하게 증가해 왔으나, 이러한 연구 동향을 체계적으로 분석한 문헌고찰 연구는 부족한 실정이며[14], 특히 디지털 성 미디어 리터러시 교육에 대한 체계적인 문헌고찰 연구는 찾아보기 힘들다. 따라서 본 연구에서는 스코핑 리뷰를 통하여 국내외 디지털 성 미디어 리터러시 교육 프로그램의 연구 동향을 파악하고, 의미 있는 결과를 분석하여 향후 청소년들에게 더욱 광범위한 영향을 미칠 것으로 예측되는 디지털 성 미디어 리터러시 연구 및 중재 프로그램 개발의 방향성을 제시하고자 한다.

### 목적

본 연구의 목적은 청소년의 디지털 성 미디어 리터러시 교육 중재 연구에 대한 스코핑 리뷰를 통하여 연구 동향을 파악하고, 연구의 범위와 주요 내용을 분석하여 향후 디지털 성 미디어 리터러시 연구의 방향을 제시하기 위함이다. 구체적인 연구 목표는 (1) 선정된 연구의 특성 평가, (2) 중재 및 결과 변수의 특성 분석, (3) 중재 효과 확인이다.

## Methods

**Ethics statement:** This study was a literature review of previously published studies and was deemed exempt from review by the Institutional Review Board of Seoul National University, as it did not involve human subjects (No. SNUIRB-2025-NH-015).

### 연구 설계

본 연구는 Arksey와 O’Malley [24]가 제안한 스코핑 리뷰 방법론 프레임워크를 기반으로 수행되었으며, (1) 연구 질문 도출, (2) 관련 문헌 검색, (3) 문헌 선정, (4) 자료 기입, (5) 결과 수집과 요약 및 보고의 5단계로 진행되었다. 연구 방법(Methods) 부분에서는 1단계부터 4단계까지를, 연구 결과(Results) 부분에서는 5단계를 서술하였다. 관련 문헌의 검색, 선정 및 종합 단계에서는 개정된 PRISMA (Preferred Reporting Items for Systematic Reviews and Meta-Analyses) 2020 가이드라인 지침을 준수하였다[25].

### 1단계: 연구 질문 도출

본 연구의 연구 질문은 “청소년의 디지털 성 미디어 리터러시 역량 강화 교육에 대한 연구의 범위와 현황은 어떠한가?”였다.

### 2단계: 관련 문헌 검색

문헌 검색은 2026년 3월 26일부터 2026년 4월 3일까지 수행되었으며, 국내외 주요 학술 데이터베이스를 이용하여 실시하였다. 국내 데이터베이스로는 RISS, KISS, DBpia, KMbase, KoreaMed를, 국외 데이터베이스로는 PubMed, CINAHL, Cochrane Library, Scopus, Web of Science를 활용하였다. 검색어는 연구 목적에 따라 한글 및 영어 키워드를 AND/OR로 조합하여 구성하였다.

한글 키워드는 “디지털,” “성 미디어 리터러시,” “성교육,” “중재,” “청소년” 등을 사용하였으며, 영어 키워드는 “digital,” “sexual media literacy,” “sexual education,” “intervention,” “adolescent” 등을 사용하였다(Supplementary Material 1). 검색을 통해 확인된 문헌의 상세 목록은 Supplementary Material 2에 제시하였다.

### 포함 및 제외 기준

2010년대 이후 스마트폰과 모바일 인터넷의 확산으로 디지털 미디어 환경이 급변하면서 성 관련 미디어와 정보 습득 수단도 달라졌다는 선행 연구[26]를 근거로, 본 연구에서는 최신 디지털 환경을 반영한 교육 프로그램의 흐름을 보다 정확히 파악하고자 최근 10년간(2015–2025) 발표된 문헌을 대상으로 분석하였다.

본 연구의 구체적인 포함 기준은 (1) 2015년 이후 발표된 연구, (2) 14–19세 청소년 대상 연구, (3) 청소년 대상 교육 전달자 또는 매개자(보건교사, 교사•상담교사, 부모/보호자)를 대상으로 한 연구, (4) 학생과 부모 또는 교사가 함께 참여한 혼합 표본 연구, (5) 성 미디어 리터러시 교육을 주제로 한 중재 연구로서 양적 연구 혹은 혼합 연구 방법론을 적용한 연구, (6) 디지털 환경(예: 소셜 미디어, 웹 기반 플랫폼, 모바일 애플리케이션 등)에서 생성 및 유통되는 성 관련 콘텐츠를 다루거나 이를 기반으로 한 교육 중재를 포함한 연구, (7) 한국어 또는 영어로 출판된 학술 문헌이다. 제외 기준은 (1) 성 관련 내용이 포함되지 않은 일반 미디어 리터러시 연구, (2) 디지털 미디어가 아닌 전통적 매체(예: 인쇄물, 오프라인 자료 등)만을 활용하여 교육한 연구, (3) 14세 미만 아동 혹은 청소년과 무관한 성인 대상의 연구, (4) 학위논문, 학술대회 초록, 보고서 등 동료 심사를 거치지 않은 회색 문헌(gray literature), (5) 중재 프로그램을 시행하지 않고 제안이나 개발 단계에서 소개만 한 연구이다.

### 3단계: 문헌 선정

**Figure 1.**
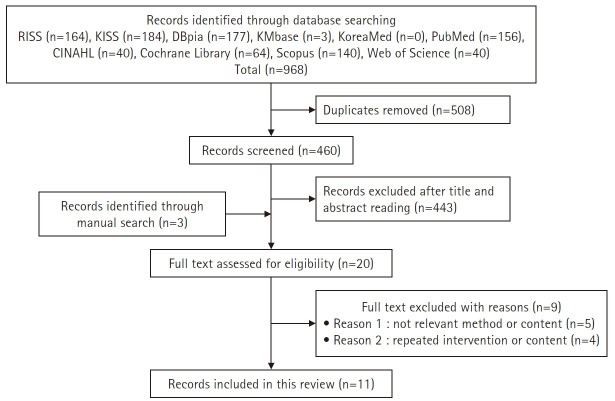
Flow chart of study selection.

검색된 문헌에서 중복을 제거한 후, 연구 목적에 근거하여 제목과 초록을 기반으로 1차 선별을 진행하였으며, 이후 원문을 정독하여 포함 및 제외 기준에 따라 최종 문헌을 선정하였다. 문헌 선정 과정은 두 명의 연구자가 독립적으로 수행하였고, 의견 불일치 시 제3의 연구자와 함께 논의를 통해 합의하였다. 선정 과정은 PRISMA 흐름도를 통해 투명하게 보고하였다(Figure 1).

### 4단계: 자료 기입

최종 선정된 문헌으로부터 다음과 같은 정보를 추출하여 표로 정리하였다. 각 문헌의 일반적 특성으로 출판 연도, 출판 국가, 출판 언어, 연구 설계 등을 포함하였다. 또한 연구 대상자의 특성을 확인하였고, 성 미디어 리터러시 교육 중재 및 프로그램의 주제와 교육 방식, 적용된 이론적 틀 및 주요 결과를 함께 분석하였다. 포함된 연구의 일반적 특성을 파악하기 위해 Excel (Microsoft, Redmond, WA, USA)을 사용하여 출판 연도, 연구 설계, 연구 국가의 빈도와 백분율을 산출하였으며, 기술통계 분석을 통해 결과를 서술하였다.

## Results

### 문헌 선정

초기 검색 결과, 총 968편의 문헌이 확인되었으며, 이 중 508편은 중복 문헌으로 제외되었다. 이후 제목과 초록을 읽은 후 443편은 선정 기준을 충족하지 않아 제외되어 17편이 남았고, 검토 과정에서 관련 논문의 인용 문헌 조사를 통해 3편의 문헌이 추가로 포함되어 20편에 대해 전문을 세밀하게 검토하였다. 이 중 5편은 실제 중재를 수행하지 않고 프로그램에 대한 제안•개발 단계에서 소개한 연구였고, 4편은 중재는 수행하였지만 동일 저자의 동일 중재 프로그램의 중복 연구로 확인되어 제외되었다. 최종적으로 11편의 문헌이 선정 기준을 충족하여 본 리뷰에 포함되었다(Figure 1).

### 선정 문헌의 일반적 특성

본 연구에서 분석한 11편의 연구 중 2010년대에 발표된 연구는 6편(54.5%), 2020년대는 5편(45.5%)이었고, 연구 수행 국가는 한국 4편(36.4%), 미국 6편(54.5%), 아일랜드가 1편(9.1%)이었다. 아울러 학문 분야를 확인하기 위하여 2025년 국가과학기술 표준분류 기준에 근거하여 분류한 결과[27], 간호과학 2편(18.2%), 아동청소년발달 4편(36.4%), 언론/미디어 정책 3편(27.3%), 교육학 3편(27.3%), 보건학 1편(9.1%), 심리과학 2편(18.2%)이었다. 연구 설계는 비동등성 대조군 전후설계(nonequivalent control group pretest-posttest design)가 5편(45.5%)으로 가장 많았고, 그다음으로는 무작위 실험연구(randomized controlled trial) 3편(27.3%), 단일집단 전후설계 연구(one-group pretest-posttest design) 3편(27.3%) 순이었다(Table 1).

### 연구 대상자 특성

연구 대상자의 특성을 분석한 결과, 청소년을 대상으로 한 연구는 7편(63.6%)이었는데, 이 중 중학생이 4편(36.4%), 고등학생은 3편(27.3%)이었다. 또한, 교사를 대상으로 한 연구는 3편(27.3%), 청소년과 부모가 모두 포함된 연구는 1편(9.1%)이었다. 성별로는 남녀 모두를 대상으로 한 연구가 8편(72.7%)으로 가장 많았다(Table 1).

### 중재 특성

#### 적용 이론 및 교육 내용

11편의 연구 중 6편(54.5%)은 이론적 틀을 적용하여 연구를 수행하였다. 적용된 이론으로는 계획된 행위 이론(theory of planned behavior, TPB)이 4편(36.4%)으로 가장 많았고, 그 다음으로는 메시지 해석 이론(message interpretation theory) 및 합리적 행동 이론(theory of reasoned action, TRA)이 각각 3편(27.3%)이었으며, 임파워먼트 이론(empowerment theory), 이중과정 설득 이론(dual-process theories of persuasion)과 3AM 모델(3AM Model [Sexual Script Acquisition, Activation and Application Model])이 각 1편(9.1%)으로 확인되었다.

디지털 성 미디어 리터러시 교육의 주요 내용을 분류한 결과, 성 미디어 해석 능력에 대한 교육이 9편(81.8%)으로 가장 많았으며, 그다음으로는 성 미디어에 대한 비판적 사고에 관한 내용 7편(63.6%), 성 미디어 리터러시 지식을 다룬 내용이 3편(27.3%)이었다. 대부분의 교육 프로그램은 단일 주제에 국한하지 않고 성 미디어 해석 능력과 비판적 사고를 동시에 포함하는 등 포괄적으로 구성되어 있어 이런 경우는 중복으로 계수하였다(Table 2).

#### 중재 기간 및 교수-학습 방법

중재 기간은 1일에서 4주까지 다양하였는데, 90–120분 이하의 4–6회기 구성이 6편(54.5%)으로 가장 많았다. 중재 효과의 지속성 확인을 위하여 추적 조사를 실시한 연구도 4편(36.4%) 있었는데, 시점은 2주에서 6개월까지였다. 사용된 교수•학습 방법은 강의 및 영상 시청을 포함한 콘텐츠 전달식 교육과 토론 및 활동지 등을 활용한 오프라인 상호 작용 활동을 병행한 중재가 7편(63.6%)으로 가장 많았다. 또한 자체 개발한 웹 기반 프로그램을 활용한 중재는 4편(36.4%)으로 나타났다(Table 2).

### 결과 변수

교육 중재의 결과 변수를 분석한 결과, 크게 지식, 태도, 행동, 신념, 리터러시 및 대인관계 과정의 6가지 유형으로 분류되었다. 교육 중재의 결과 변수를 성 건강에 대한 지식으로 측정한 연구는 총 4편(36.4%)으로 4편 모두에서 교육 후 지식이 유의하게 향상되었다. 또한 태도 영역의 효과를 측정한 연구는 총 6편(54.5%)이었는데, 이 중 성 태도를 측정한 2편의 연구와 데이트 폭력 태도에 대한 2편 중 1편의 연구는 교육 후 긍정적인 효과를 보였다. 또한 성 인지 감수성, 성 역할에 대한 태도, 교사의 청소년에 대한 이중잣대 태도 및 성행위 지연 태도를 결과 변수로 측정한 연구들이 각각 1편씩 수행되었으며 교육 후 모두 향상되었다.

행동 영역의 효과를 측정한 연구는 총 5편(45.5%)이었는데, 이 중 성행위 의도를 결과 변수로 측정한 연구가 3편이었다. 성행위 의도의 하위 변수로 성관계, 피임 및 생식건강 행위를 측정하였는데, 이 중 피임과 생식건강 행위는 교육 후 긍정적인 효과를 보였으나 성관계는 통계적으로 유의한 변화가 없었다. 1편의 연구에서 성행위 지연에 대한 예측 및 성행위 지연 효능감을 측정하였는데, 성행위 지연에 대한 예측은 교육 후 긍정적인 효과를 보였으나, 성행위 지연 효능감은 통계적으로 유의한 효과를 보이지 않았다. 또한 다른 1편의 연구에서는 성적 동의와 성적 의사결정에 대해 측정하였는데, 성적 동의는 교육 후 긍정적인 효과를 보였으나, 성적 의사 결정에서는 성별에 따른 차이를 보였다. 또한 규범적 신념 및 생식기 자기 이미지(genital self-image)와 같은 신념 영역의 효과를 측정한 연구는 총 2편(18.2%)이었는데, 모두 긍정적인 효과를 보였다.

교육 중재의 효과로 리터러시를 변수로 측정한 연구는 총 8편(72.7%)이었는데, 이 중 1편을 제외하고는 모두 성 미디어 리터러시에 대한 긍정적 교육 효과를 나타냈다. 한편, 교육이 대인관계 과정에 미치는 효과를 측정한 연구도 총 4편(36.4%)이었는데, 이들은 모두 성 건강 의사소통을 효과 변수로 측정하고 있었고, 추가로 부모-청소년 유대감도 포함하였다. 성 건강 의사소통 연구 4편 중 2편과 부모-청소년 유대감을 측정한 연구는 교육 후 통계적으로 유의한 변화를 보였으나, 나머지 2편에서는 변화가 보고되지 않았다(Table 3).

## Discussion

본 연구는 최근 10년간 출판된 청소년의 디지털 성 미디어 리터러시 교육 중재의 연구 동향을 파악하고 특성을 분석하여, 디지털 성 미디어 리터러시 교육과 연구의 바람직한 방향을 제시하고자 하였다. 먼저 선정 문헌의 일반적인 특성을 살펴보면, 최근 10년간 관련 연구가 꾸준히 진행된 것을 볼 수 있는데, 최근 청소년 대상 디지털 성 미디어 리터러시의 중요성이 강조됨에 따라 향후 이 분야의 연구도 더욱 증가할 것으로 예상된다. 연구 수행 지역은 미국이 6편으로 가장 많아, 미국에서 청소년 성교육과 미디어 리터러시 교육에 대한 연구가 활발히 이루어지고 있음을 알 수 있다.

연구 설계는 비동등성 대조군 전후설계가 가장 많았으며, 국내 연구의 경우 모든 연구가 해당 설계를 적용하여 프로그램의 효과성을 명확히 입증하는 데는 한계가 있다고 볼 수 있다[28]. 반면 국외 연구는 무작위 대조군 실험설계로 보다 엄격한 방법론적 접근으로 수행되어, 향후 국내 연구에서도 프로그램의 효과를 보다 정확하게 검증할 수 있도록 무작위 배정 등 실험 설계의 강화가 필요함을 시사한다. 국가과학기술 표준분류 기준[27]에 근거한 학술 분야별로는 간호과학을 비롯한 교육학, 심리과학, 아동청소년발달, 언론/미디어 정책, 보건학 등 다양한 분야에서 연구가 수행되었고, 다학문적 접근도 이루어지고 있음을 알 수 있었다.

연구 대상자를 분석한 결과, 중학생이 다수였다. 우리나라 청소년들의 성관계 시작 연령은 평균 13세로 보고된 바 있는데[29], 이는 중학생 시점으로, 이 시기는 발달 특성상 이성에 대한 관심과 성적 호기심이 증가하는 시기이다[30]. 특히, 우리나라 청소년들은 스마트폰과 인터넷 보급률이 높아 성 관련 미디어에 노출되기 쉬운 환경에 처해 있어[29], 왜곡된 성 정보에 대한 접촉은 부정적인 결과를 초래할 수 있다[31]. 따라서 중학생을 대상으로 하는 미디어 리터러시 교육은 올바른 성 가치관 형성을 위한 필수적인 개입으로 정규 교육과정에 포함되어야 할 것이다.

교육 프로그램의 구성에서 사용된 이론적 틀을 살펴보면, TPB 이론이 가장 많이 활용되었는데, 선행 연구에 따르면 TPB 이론을 기반으로 설계된 교육이 청소년의 학습 증진을 높여 학습 의도에 긍정적인 영향을 미쳤다고 한다[32]. 메시지 해석 이론과 TRA 이론이 두 번째로 많이 사용되었다. 메시지 해석 이론에 의하면 메시지 처리 수준이 높을수록 청소년의 긍정적 성 행동 의도가 증가한다는 연구 결과도 보고되어, 청소년 성 행동과 관련한 미디어 메시지 처리 능력이 중요한 역할을 하는 것으로 나타났다[33]. TRA 이론은 개인의 태도와 주관적 규범이 행동 의도를 형성하고, 이는 실제 행동으로 이어지기 때문에, TRA 기반 성교육 중재는 성 관련 태도와 행동을 변화시키고 고위험 성 행동을 감소시키는 데 효과적이라고 보고하였다[34]. 따라서 청소년의 디지털 성 미디어 리터러시 교육 중재 프로그램에 계획된 행동 이론, 메시지 해석 이론 및 합리적 행동 이론을 적용하여 구성하는 것은 바람직하다고 볼 수 있다.

교육 주제를 분석한 결과, 디지털 성 미디어 리터러시를 높이기 위하여 미디어를 비판적으로 해석하고 분석하는 능력을 강화하는 내용이 가장 많았다. 최근 딥페이크, 가짜 뉴스 등 비사실적 정보가 양산되는 환경에서[9], 청소년들에게 이러한 정보를 올바르게 분별할 수 있는 능력을 갖출 수 있도록 하는 교육 프로그램의 개발과 보급이 확산되어야 한다. 교육 운영 시간은 1회기당 90–120분 이하로 평균 4–6회기에 걸쳐 진행하는 수업이 많았다. 이는 청소년의 집중력과 학습 효과의 지속성 유지를 고려한 설계로 바람직하다고 볼 수 있다. 반면, 부모를 대상으로 한 연구는 웹 기반 프로그램을 통해 자율적으로 학습하도록 구성하여, 성인 학습자의 유연한 학습 환경을 제공한 것이 특징적이었다. 또한 교사 대상 연구에서는 짧은 기간 내에 집약적으로 교육(총 15–18시간)하는 방식을 사용하였는데, 이는 교사들의 제한된 시간과 교육 일정에 맞춘 대상자 맞춤형 설계라 볼 수 있다. 이러한 결과는 교육 대상과 특성에 따라 중재 운영 방식이 달라져야 하며, 대상 맞춤형 설계가 교육 효과를 높이는 중요한 요인임을 시사한다.

교수-학습 방법으로는 온라인 영상을 활용한 콘텐츠 전달식 강의와 오프라인에서 토론을 통한 상호작용 활동을 병행한 교육이 가장 많았는데, 이는 전통적인 일방향의 전달식 학습법을 지양하고 활동지 작성, 발표, 토론 등을 통해 대상자의 적극적인 참여를 유도하기 위함이다. 또한 웹 기반 프로그램을 개발하여 이를 활용한 중재도 4편으로 확인되었는데, 콘텐츠 전달 중심이 아닌 참여형 학습 전략을 적용하여 대상자의 적극적인 참여를 유도하고자 설계하였다. 즉, 참여자들은 성적 미디어의 생산과 재현 방식을 이해하고 이를 비판적으로 분석하는 활동에 참여함으로써, 디지털 미디어의 성적 규범과 신체 이미지에 대한 왜곡 과정을 인식하도록 구성하였다. 또한 의사소통 기술에 대한 학습 내용과 실제 성적 관계에서의 건강한 의사결정 과정도 포함하여 바람직하다고 볼 수 있다.

청소년을 대상으로 한 교육에서 다양한 교육 매체를 활용하여 학생에게 맞춘 개별화 수업은 학업 성취도, 효능감, 흥미를 높인다는 연구 결과가 보고된 바 있다[35]. 특히 오늘날과 같은 지식 정보화 사회에서는 정보 변화의 가속화와 지식의 폭발적인 증가로 인하여 교육 방법에도 큰 변화가 요구되어, 기존의 교수자 중심 교육법이 아닌 학습자 중심 교육으로 전환하고자 하는 노력이 필요하다[36]. 따라서 청소년의 디지털 성 미디어 리터러시 교육에서 발표 및 토론과 같은 참여형 교육과 더 나아가 실제 미디어를 활용한 실천형 교육을 확대할 필요가 있다.

각 연구에서 보고된 중재 효과를 체계적으로 정리하기 위해 연구에서 사용한 원 개념을 최대한 존중하여 결과 변수를 분류하였다. 문헌 리뷰 결과, 중재 결과 변수는 지식, 태도, 행동, 신념, 리터러시 및 대인관계 과정의 6가지 영역으로 분류되었다.

성 건강에 대한 지식을 결과 변수로 측정한 연구는 4편 모두에서 교육 후 지식이 유의하게 향상되어 청소년이 성 건강 지식을 얻는 데 있어 교육적 중재가 효과적인 것으로 나타났다. 선행 연구에서도 성교육 중재를 받은 청소년은 중재 직후뿐만 아니라 1년 후까지도 성 지식에서 유의한 향상을 보인 바 있다[37]. 그러나 본 연구에서 고찰된 문헌들은 대부분 양적 단회성 효과 측정에 그쳤으며, 급변하는 디지털 성 미디어 환경에서 청소년들이 올바른 지식을 체화하기 위해서는 효과의 지속성이 담보되어야 한다. 따라서 향후 연구에서는 중재 효과의 지속성을 검증하는 종단 연구가 필요하다.

태도를 측정한 연구에서도 교육 중재의 효과가 확인되어 청소년이 성 건강의 중요성을 인식하고 올바른 태도를 가질 수 있도록 교육적 개입이 필요함을 시사한다. 선행 연구에서도 성교육 중재를 받은 청소년은 중재 후 성 태도에서 장기적으로 긍정적인 효과를 보인 바 있다[37]. 한편, 데이트 폭력 태도의 중재 효과에서 두 편의 연구 중 한 편은 효과를 보이지 않았는데, 해당 연구를 분석해 보면 통계적으로 유의한 차이는 아니지만, 남학생에게서 중재 후 측정값이 증가하였다. 이는 남학생이 여학생보다 성폭력 가해 위험성이 높기 때문에[38], 가해자인 남학생에게서 직접적인 효과가 나타난 것으로 추측되며, 추후 성별에 따른 차이를 검증하기 위한 후속 연구가 필요하다.

교육이 청소년의 성 행동에 미치는 효과에서는 교육 후 성행위 지연에 대한 예측과 성행동전 동의에서는 긍정적인 변화를 보였으나 성행위 지연 효능감에서는 효과를 보이지 않았고, 성적 의사결정에서는 성별에 따라 효과가 다르게 나타났다. 또한 성행위 의도는 하위변수에 따라 상이한 결과를 보였는데, 피임은 교육 후 긍정적인 효과를 보였으나 성관계는 통계적으로 유의한 변화가 없었다. 부모를 대상으로 한 청소년 성 건강 중재 메타분석 연구에서도 지식이나 태도에 비해 행동적 변화는 장기간에 걸친 중재에서 비로소 효과를 보인다고 하였다[39]. 이는 청소년의 바람직한 성 행동 변화를 위해서는 장기에 걸친 체계적인 교육과 개입이 필요함을 시사하며, 추후 행동과 같은 결과의 차이를 검증하기 위하여 대상자의 성별과 연령 등 인구학적 특성과 함께 교육 기간을 고려한 장기적 중재와 지속적인 추적 연구가 필요할 것으로 판단된다.

규범적 신념 및 생식기 자기 이미지를 측정한 연구 모두에서도 통계적으로 유의한 중재 효과가 확인되었으며, 선행 연구에서도 교육적 중재를 통해 성 건강에 대한 규범적 신념 등이 향상될 수 있음이 보고되었다[40]. 규범적 신념을 측정한 분석 대상 문헌에 따르면, 디지털 성 미디어 리터러시 중재는 미디어 속에서 과장되게 묘사되는 위험한 성 행동을 청소년이 객관적으로 인지하도록 도움으로써, 특히 '묘사적 규범 신념(descriptive norms)'을 긍정적으로 변화시키는 것으로 나타났다[41]. 또한, 이러한 중재 효과가 청소년의 성별에 따라 차이가 있음이 함께 확인되었지만[41], 현재까지는 성별 차이가 발생하는 구체적인 기제와 원인이 명확히 규명되지 않은 실정이다. 따라서 향후 연구에서는 청소년의 성별에 따른 디지털 성 미디어 신념 형성 과정을 고려한 성별 맞춤형 디지털 성 미디어 리터러시 중재 연구가 수행되어야 할 것이다.

교육이 청소년의 디지털 성 미디어 리터러시에 미치는 효과를 분석한 연구에서는 8편 중 7편에서 긍정적인 효과를 보고하였다. 이를 통하여 교육 중재는 청소년이 성적 미디어 콘텐츠를 올바른 정보와 가치관에 기반하여 비판적으로 해석하고 판단할 수 있는 역량을 강화할 수 있음을 시사한다. 한편, 성 미디어 리터러시 연구 중 유일하게 효과를 보이지 않았던 Dodson 등[42]의 연구를 분석한 결과, 교육 중재 1개월 후에 측정한 단기 효과는 확인되었으나, 6개월에 걸친 추적 평가에서는 청소년의 미디어 관련 효과가 유지되지 않았다. 이는 부모를 중재 대상자로 삼아 청소년의 변화를 간접적으로 유도하는 매개 중재의 경우, 청소년의 변화를 장기적으로 지속하는 데 일정 부분 한계가 있음을 시사한다. 따라서 청소년의 디지털 성 미디어 리터러시를 향상하고 그 효과를 장기적으로 유지하기 위해서는 부모 교육과 더불어 청소년을 직접적인 대상으로 포함하여 병행하는 접근이 요구된다. 아울러 시간이 경과함에 따라 교육 효과가 약화되는 것을 방지하기 위해 중재 이후에도 주기적인 보수 교육을 통한 지속적인 중재 시스템 구축이 바람직할 것이다.

대인관계 과정에 미치는 효과를 측정한 연구에서는 성 건강 의사소통과 부모-청소년 유대감을 결과 변수로 포함하였다. 먼저, 성 건강 의사소통 능력은 4편 중 2편만이 효과를 보였는데, 선행 연구에서도 부모-청소년 간 성 의사소통이 청소년의 피임 및 콘돔 사용과 같은 안전한 성 행동에 긍정적인 영향을 준다고 보고하였으며[43], 이는 부모-자녀 대화가 성적 의사결정 과정에서 보호 요인으로 작용할 수 있음을 시사한다. 또한 부모와의 유대감이 높을수록 성 건강 관련 대화가 보다 원활히 이루어지고, 성 관련 문제를 상의하는 빈도 역시 증가하는 것으로 나타나[44] 부모와 자녀 간 관계의 질이 자녀의 성 건강을 위한 의사소통을 촉진할 수 있음을 보여준다. 한편, 교육의 효과를 보이지 않은 두 편의 연구를 살펴보면, 먼저 Dodson 등[42]의 연구에서는 중재 후 1개월 시점에서는 의사소통 효과가 확인되었으나, 6개월 이후까지 장기적으로 유지되지 못하는 한계가 있었다. 즉, 중재 후 초기에는 부모와 자녀 간의 성 관련 대화가 활성화되었으나 시간이 지남에 따라 약화되었기 때문에, 의사소통 효과의 지속성 확보를 위해서는 반복적이고 장기적인 개입이 필요함을 알 수 있다. 또한 Scull 등[41]의 연구에서는 성별에 따른 차이를 보고하였는데, 중재 후 여학생은 부모와의 의사소통 빈도가 증가한 반면, 남학생은 효과가 미미하였다. 연구자들은 이러한 결과에 대해 성 건강 관련 의사소통이 주로 어머니 중심으로 이루어지는 경향이 있어 여학생이 부모와의 성 관련 대화에 보다 익숙하게 반응했을 가능성을 제시하였다. 반면 남학생의 경우, 남성은 성 건강과 같은 민감한 주제를 드러내어 논의하지 않는다는 성별 고정관념이 의사소통에 영향을 미쳤을 가능성이 있다고 해석하였다[41]. 이는 선행 연구의 결과와도 유사한 맥락을 보이는데, Widman 등[43]은 일부 아버지와 남학생이 감정적 경험이나 성 관련 주제를 개방적으로 논의하는 데 어려움을 느꼈으며, 이러한 성별 특성이 성 건강 관련 의사소통을 제한할 가능성이 있다고 설명하였다. 따라서 추후 연구에서는 성별에 따른 맞춤형 의사소통 증진 전략이 추가적으로 필요할 것이다.

### 결론

본 연구를 통하여 디지털 성 미디어 리터러시 교육은 청소년의 성 지식, 태도, 행동, 리터러시, 신념 및 대인관계 과정에 긍정적인 영향을 미칠 수 있음을 확인하였다. 또한 청소년 당사자뿐 아니라 부모와 교사를 대상으로 한 교육적 중재도 청소년의 디지털 성 미디어 리터러시 증진에 효과가 있었다. 따라서 청소년은 물론 부모, 교사 등 주요 양육자와 교육자의 적극적인 참여를 유도하여, 가정과 학교를 포함한 사회 전반에 걸친 청소년의 성 건강 증진을 위한 지지적 환경을 조성할 필요가 있다.

본 연구는 2010년대 이후 디지털 미디어 환경이 급변하면서 성적 미디어와 정보 습득 수단도 달라졌다는 선행 연구의 주장에 근거하여[26] 최신 디지털 환경을 반영한 교육 프로그램의 흐름을 보다 정확히 파악하고자 최근 10년간의 연구를 대상으로 하였다. 영어와 한국어로 출판된 논문만을 분석 대상으로 추출하였기 때문에 타 언어권에서 수행된 연구는 배제되었을 가능성도 있다. 아울러 분석에 포함된 중재들은 프로그램 내용, 전달 방식, 연구 설계 및 결과 측정 방법 등에서 차이를 보여 중재 효과를 직접적으로 비교하는 데는 한계가 있었다. 따라서 본 연구 결과를 일반화하는 것에는 신중한 해석이 필요하다.

그럼에도 불구하고 본 연구는 최근 우리 사회에서 중요한 현안 과제로 대두된 청소년의 무분별한 성 미디어 노출 문제를 해소하고자 최초로 청소년의 디지털 성 미디어 리터러시 교육 중재의 국내외 연구 동향을 포괄적으로 분석하고 정리하였다는 점에서 매우 의의가 있다. 특히 교육 프로그램 개발의 근거 이론, 교육 내용, 교육 기간, 교수-학습 방법 및 결과 변수 등을 다각도로 분석함으로써 향후 디지털 성 미디어 리터러시 교육 중재 프로그램 설계의 기초 자료로도 활용될 수 있을 것으로 기대된다.

## Figures and Tables

**Table 1.** General characteristics of the selected studies and their participants (N=11)

Variable	Category	n (%)
Publication year	2015–2019	6 (54.5)
	2020–2025	5 (45.5)
Country	Korea	4 (36.4)
	USA	6 (54.5)
	Ireland	1 (9.1)
Publication language	Korean	3 (27.3)
	English	8 (72.7)
Academic discipline (mixed use)	Nursing science	2 (18.2)
	Child and adolescent development	4 (36.4)
	Media and communication policy	3 (27.3)
	Education	3 (27.3)
	Public health	1 (9.1)
	Psychology	2 (18.2)
Study design	Nonequivalent control group pretest-posttest design	5 (45.4)
	Randomized controlled trial design	3 (27.3)
	One-group pretest-posttest design	3 (27.3)
Subjects	Adolescents	7 (63.6)
	Middle school	4 (36.4)
	High school	3 (27.3)
	Teachers	3 (27.3)
	Adolescents+parents	1 (9.1)
Gender of subjects	Female+male	8 (72.7)
	Not provided	3 (27.3)

**Table 2.** Characteristics of the sex education programs in selected studies (N=11)

Variable	Category	n (%)
Theoretical framework (mixed use)	Theory of planned behavior	4 (36.4)
	Message interpretation theory	3 (27.3)
	Theory of reasoned action	3 (27.3)
	Empowerment theory	1 (9.1)
	Dual-process theories of persuasion	1 (9.1)
	3AM Model	1 (9.1)
Main theme in sexual media literacy education (mixed use)	Interpretation of sexual media	9 (81.8)
	Critical thinking about sexual media	7 (63.6)
	Knowledge of sexual media literacy	3 (27.3)
Education period (duration/sessions)	45 min, 4 times	1 (9.1)
	45 min, 5 times	1 (9.1)
	45 min, 6 times	1 (9.1)
	50 min, 6 times	1 (9.1)
	90 min, 1 time	1 (9.1)
	90–120 min, 5 times	1 (9.1)
	100 min, 9 times	1 (9.1)
	2 hours/9 times	1 (9.1)
	4 hours/1 time	1 (9.1)
	Not provided/10 times	1 (9.1)
	Self-paced	1 (9.1)
Follow-up timing	2 weeks later	1 (9.1)
	4 weeks later	1 (9.1)
	3 months later	1 (9.1)
	6 months later	1 (9.1)
Teaching methods (mixed use)	Lecture-based delivery	7 (63.6)
	(content delivery through lectures or video viewing)	
	Web-based program (researcher-developed web-based intervention)	4 (36.4)
	Offline interactive activities	7 (63.6)
	(discussion, worksheets, and other in-person activities)	

3AM Model: Sexual Script Acquisition, Activation and Application Model.

**Table 3.** Effects of sexual media literacy programs (N=11)

Category	Total	Variable	n (%)	No. of effective studies
Knowledge	4	Sexual health knowledge [A4,6,7,10]	4 (36.4)	4 [A4,6,7,10]
Attitude	6	Sexual attitude [A6,7]	2 (18.2)	2 [A6,7]
		Gender sensitivity [A7]	1 (9.1)	1 [A7]
		Gender role attitude [A4]	1 (9.1)	1 [A4]
		Dating violence attitude [A4,10]	2 (18.2)	1 [A4]
		Double standard attitude [A9]	1 (9.1)	1 [A9]
		Delaying sexual activity attitude [A1]	1 (9.1)	1 [A1]
Behavior	5	Sexual behavior intentions [A4,8,10]	3 (27.3)	1 [A4]
		Sexual intercourse [A4,8,10]	3 (27.3)	0
		Contraceptives [A4,10]	2 (18.2)	1 [A4]
		Expectation of delaying sexual activity [A1]	1 (9.1)	1 [A1]
		Self-efficacy of delaying sexual activity [A1]	1 (9.1)	0
		Sexual consent preparedness [A11]	1 (9.1)	1 [A11]
		Sexual decision making [A11]	1 (9.1)	1 [A11]
Belief	2	Normative beliefs [A10]	1 (9.1)	1 [A10]
		Genital self-image [A11]	1 (9.1)	1 [A11]
Literacy	8	Sexual media literacy [A2–5,8–11]	8 (72.7)	7 [A2–5,9–11]
Interpersonal process	4	Sexual health communication [A4,8–10]	4 (36.4)	2 [A4,9]
		Parent-adolescent bonding [A8]	1 (9.1)	1 [A8]

## Supplementary materials

Further details on supplementary materials are presented online (available at https://doi.org/10.4069/whn.2026.05.26).

Supplementary Material 1. Literature search strategy

Supplementary Material 2. List of studies included in the scoping review
