# Supplementary material for: Research trends in educational interventions for digital sexual media literacy among adolescents: a scoping review (2015–2025)
Source: Womens Health Nurs. 2026 Jun 30;32(2):93–103. [Article in Korean] doi: 10.4069/whn.2026.05.26 (PMC13346788; doi:10.4069/whn.2026.05.26)
Supplement: Supplementary Material 1. — Literature search strategy [file whn-2026-05-26-Supplementary-Material-1.pdf]

Supplementary Material 1. Literature search strategy

1. 검색 개요

본 연구는 '청소년 디지털 성 미디어 리터러시'에 관한 문헌을 검색하기 위해 국내외 10개의 학술 데이터베이스를 활용하였다. 검색 기간은 2015년부터 2025년 12월 30일까지로 설정하였다.

2. 데이터베이스 목록

1) 국내 데이터베이스

- 학술연구정보서비스(Research Information Sharing Service, RISS)
- 누리미디어(Data Base Periodical Information Academic, DBpia)
- 한국학술정보(Korean studies Information Service System, KISS)
- KMbase
- KoreaMed

2) 국외 데이터베이스

- PubMed
- CINAHL
- Cochrane Library
- Scopus
- Web of Science

3. 검색어 구성

1) 국내 데이터베이스 검색어 구성

Concept	Search terms
Concept 1 (digital)	디지털 OR 온라인 OR 인터넷 OR 모바일 OR 스마트폰 OR 소셜미디어
Concept 2 (media literacy)	미디어 리터러시 OR 디지털 리터러시 OR 디지털 미디어 리터러시
Concept 3 (sexual)	성 OR 성교육 OR 성정보 OR 성콘텐츠
Concept 4 (intervention/education)	교육 OR 중재 OR 프로그램
Concept 5 (population)	청소년 OR 중학생 OR 고등학생

(미디어 리터러시와 디지털 리터러시는 개념적으로 구분되나, 실제 연구에서는 혼용되어 사용되는 경우가 많아 관련 문헌의 포괄적 수집을 위해 검색 단계에서는 두 용어를 모두 포함하였다.)

https://doi.org/10.4069/whn.2026.05.26

2) 국외 데이터베이스 검색어 구성

Concept	Controlled vocabulary (MeSH/Thesaurus)	Free text/keywords
Concept 1 (digital)	"Internet"[MeSH]<br>"Cell Phone"[MeSH]<br>"Social Media"[MeSH]<br>"Computers, Handheld"[MeSH]	digital OR online OR internet OR mobile OR smartphone OR "social media"
Concept 2 (media literacy)	"Media Literacy"[MeSH]	"media literacy" OR "digital literacy" OR "digital media literacy"
Concept 3 (sexual)	"Sex Education"[MeSH]<br>"Sexual Health"[MeSH]<br>"Pornography"[MeSH]	sex OR sexual OR "sex education" OR "sexual health" OR pornography
Concept 4 (intervention/education)	"Health Education"[MeSH]<br>"Program Evaluation"[MeSH]	education OR intervention OR program OR training
Concept 5 (population)	"Adolescent"[MeSH]	adolescent OR teenager OR youth OR "middle school student*" OR "high school student*"

(*: 복수형, " ": 한 구문으로 인식)

4. 검색어 조합 및 전략

본 연구에서는 디지털 환경, 미디어 리터러시, 성 관련 개념, 중재/교육, 대상자(청소년)를 포함한 5가지 핵심 개념을 기반으로 검색 전략을 수립하였다. 그러나 예비 검색(pilot search) 결과, 모든 개념을 동시에 적용할 경우 검색 결과가 과도하게 제한되어 충분한 문헌 추출에 한계가 있었다. 이에 따라 검색의 민감도를 높이기 위해 핵심 개념인 디지털, 미디어 리터러시, 성 관련 개념을 중심으로 검색식을 구성하였다.

1) 국내 데이터베이스 검색 전략

국내 DB 기본 검색식

(디지털 OR 온라인 OR 인터넷 OR 모바일 OR 스마트폰 OR 소셜미디어)
AND
(미디어 리터러시 OR 디지털 리터러시)
AND
(성 OR 성교육 OR 성정보 OR 성콘텐츠)

공통 필터: 학술지, 2015–2025년

검색 결과

(1) RISS (검색일: 2026년 4월 1일)
- 검색필드: 전체
- 검색결과: 164건

(2) KISS (검색일: 2026년 4월 1일)
- 검색필드: 전체
- 제한조건: 미등재
- 검색결과: 184건

(3) DBpia (검색일: 2026년 4월 1일)
- 검색필드: 전체
- 검색결과: 177건

https://doi.org/10.4069/whn.2026.05.26

(4) KMbase (검색일: 2026년 4월 1일)
- 검색필드: 전체
- 검색결과: 3건

(5) KoreaMed (검색일: 2026년 4월 1일)
- 검색필드: 전체
- 검색결과: 0건

2) 국외 데이터베이스 검색 전략

공통 필터: 영어, 연구 논문(Article), 2015–2025년

검색 결과

(1) PubMed (검색일: 2026년 4월 2일)
- 검색어 조합:

((("Internet"[MeSH Terms]) OR ("Cell Phone"[MeSH Terms]) OR ("Social Media"[MeSH Terms]) OR ("Computers, Handheld"[MeSH Terms]) OR ("digital"[Title/Abstract]) OR ("online"[Title/Abstract])
OR ("internet"[Title/Abstract]) OR ("mobile"[Title/Abstract]) OR ("smartphone"[Title/Abstract]) OR ("social media"[Title/Abstract]))
AND
(("Media Literacy"[MeSH Terms]) OR ("media literacy"[Title/Abstract]) OR ("digital literacy"[Title/Abstract]) OR ("digital media literacy"[Title/Abstract]))
AND
(("Sex Education"[MeSH Terms]) OR ("Sexual Health"[MeSH Terms]) OR ("Pornography"[MeSH Terms]) OR ("sex"[Title/Abstract]) OR ("sexual"[Title/Abstract]) OR ("sex education"[Title/Abstract]) OR ("sexual health"[Title/Abstract]) OR ("pornography"[Title/Abstract]))
AND
(("Health Education"[MeSH Terms]) OR ("Program Evaluation"[MeSH Terms]) OR ("education"[Title/Abstract]) OR ("intervention"[Title/Abstract]) OR ("program"[Title/Abstract]) OR ("training"[Title/Abstract]))
AND
(("Adolescent"[MeSH Terms]) OR ("adolescent"[Title/Abstract]) OR ("teenager"[Title/Abstract]) OR ("youth"[Title/Abstract]) OR ("middle school student*"[Title/Abstract]) OR ("high school student*"[Title/Abstract]))
- 필터: 인간 대상 연구
- 검색결과: 156건

(2) CINAHL (검색일: 2026년 4월 2일)
- 검색어 조합:

((MH "Internet") OR TI digital OR AB digital OR TI online OR AB online OR TI internet OR AB internet OR TI mobile OR AB mobile OR TI smartphone OR AB smartphone OR TI "social media" OR AB "social media" OR (MH "Social Media") OR (MH "Cell Phone") OR (MH "Computers, Handheld"))
AND
((MH "Media Literacy") OR TI "media literacy" OR AB "media literacy" OR TI "digital literacy" OR AB "digital literacy" OR TI "digital media literacy" OR AB "digital media literacy")
AND
((MH "Sex Education+") OR (MH "Sexual Health+") OR (MH "Pornography") OR TI sex OR AB sex
OR TI sexual OR AB sexual OR TI "sex education" OR AB "sex education" OR TI "sexual health" OR AB "sexual health" OR TI pornography OR AB pornography)
AND
((MH "Health Education+") OR (MH "Program Evaluation") OR TI education OR AB education OR TI intervention OR AB interven-

tion OR TI program OR AB program OR TI training OR AB training)

AND

((MH "Adolescence+") OR TI adolescent OR AB adolescent OR TI teenager OR AB teenager OR TI youth OR AB youth OR TI "middle school student*" OR AB "middle school student*" OR TI "high school student*" OR AB "high school student*")

- 검색결과:40건

(3) Cochrane Library (검색일: 2026년 4월 2일)

- 검색어 조합: (digital OR online OR internet OR mobile OR smartphone OR "social media")

AND

("media literacy" OR "digital literacy" OR "digital media literacy")

AND

(sex OR sexual OR "sex education" OR "sexual health" OR pornography)

AND

(education OR intervention OR program OR training)

AND

(adolescent OR teenager OR youth)

- 검색결과: 64건

(4) Scopus (검색일: 2026년 4월 2일)

- 검색어 조합:

(TITLE-ABS-KEY (digital OR online OR internet OR mobile OR smartphone OR "social media")

AND TITLE-ABS-KEY ("media literacy" OR "digital literacy" OR "digital media literacy")

AND TITLE-ABS-KEY (sex OR sexual OR "sex education" OR "sexual health" OR pornography)

AND TITLE-ABS-KEY (education OR intervention OR program OR training)

AND TITLE-ABS-KEY (adolescent OR teenager OR youth OR "middle school student*" OR "high school student*"))

- 검색결과: 140건

(5) Web of Science (검색일: 2026년 4월 2일)

- 검색어 조합:

(TS=(digital OR online OR internet OR mobile OR smartphone OR "social media")

AND TS=("media literacy" OR "digital literacy" OR "digital media literacy")

AND TS=(sex OR sexual OR "sex education" OR "sexual health" OR pornography)

AND TS=(education OR intervention OR program OR training)

AND TS=(adolescent OR teenager OR youth OR "middle school student*" OR "high school student*"))

- 검색결과: 40건

https://doi.org/10.4069/whn.2026.05.26
