# Supplementary material for: Research trends in educational interventions for digital sexual media literacy among adolescents: a scoping review (2015–2025)
Source: Womens Health Nurs. 2026 Jun 30;32(2):93–103. [Article in Korean] doi: 10.4069/whn.2026.05.26 (PMC13346788; doi:10.4069/whn.2026.05.26)
Supplement: Supplementary Material 2. — List of studies included in the scoping review [file whn-2026-05-26-Supplementary-Material-2.pdf]

Supplementary Material 2. List of studies included in the scoping review

A1. Austin EW, Pinkleton BE, Chen YC, Austin BW. Processing of sexual media messages improves due to media literacy effects on perceived message desirability. Mass Commun Soc. 2015;18(4):399-421. https://doi.org/10.1080/15205436.2014.1001909

A2. Baek S, Jun E, Kwon I, Lee KH, Lee JE. The effects of media literacy-focused sex education program on teachers. J Korean Soc Sch Health. 2015;28(3):229-238. https://doi.org/10.15434/kssh.2015.28.3.229

A3. Rothman EF, Adhia A, Christensen TT, Paruk J, Alder J, Daley N. A pornography literacy class for youth: results of a feasibility and efficacy pilot study. Am J Sex Educ. 2018;13(1):1-17. https://doi.org/10.1080/15546128.2018.1437100

A4. Scull TM, Kupersmidt JB, Malik CV, Morgan-Lopez AA. Using media literacy education for adolescent sexual health promotion in middle school: randomized control trial of Media Aware. J Health Commun. 2018;23(12):1051-1063. https://doi.org/10.1080/10810730.2018.1548669

A5. Baek SS, Min HY, Lee JE, Kim SJ. Effects of sexual media literacy education for school nurses in South Korea. J Sch Nurs. 2019;35(4):268-278. https://doi.org/10.1177/1059840518758377

A6. Kim YH, Choi E, Kim C. The effect of sexuality education program using media literacy on sexual knowledge and sexual knowledge of middle school students. Stud Korean Youth. 2020;27(7):237-256. https://doi.org/10.21509/kjys.2020.07.27.7.237

A7. Park YW, Seo EH. The effects of a sex education program using media literacy for adolescents. J Learn Cent Curric Instr. 2021;21(13):201-215. https://doi.org/10.22251/jlcci.2021.21.13.201

A8. Dodson CV, Scull T, Schoemann AM. A six-month outcome evaluation of Media Aware Parent, a parent-based media mediation and sexual health communication program to promote adolescent sexual health. J Health Commun. 2022;27(11-12):825-838. https://doi.org/10.1080/10810730.2023.2165741

A9. Maas MK, Gal T, Cary KM, Greer K. Popular culture and pornography education to improve the efficacy of secondary school staff response to student sexual harassment. Am J Sex Educ. 2022;17(4):435-457. https://doi.org/10.1080/15546128.2022.2076757

A10. Scull TM, Dodson CV, Geller JG, Reeder LC, Stump KN. A media literacy education approach to high school sexual health education: immediate effects of Media Aware on adolescents' media, sexual health, and communication outcomes. J Youth Adolesc. 2022;51(4):708-723. https://doi.org/10.1007/s10964-021-01567-0

A11. Dawson K, Sharp G, O'Higgins S, McIvor C, Macneela P. Evaluation of a sex-positive, youth-centred online pornography literacy intervention for adolescents. Sex Educ. 2025 Sep 9 [Epub]. https://doi.org/10.1080/14681811.2025.2541633
